# Spontaneous Curvature Sculpts Filament–Vesicle Morphogenesis

**DOI:** 10.1021/acs.nanolett.6c02160

**Published:** 2026-07-23

**Authors:** Yaxin Fang, Chengyao Zhang, Meng Wang, Chao Shi, Xin Yi

**Affiliations:** † School of Mechanics and Engineering Science, 12465 Peking University, Beijing 100871, China

**Keywords:** filament−vesicle coupling, spontaneous curvature, vesicle confinement, curvature elasticity, symmetry breaking, artificial cells

## Abstract

Membrane-bound filaments are central to cellular morphogenesis, yet how spontaneous curvature regulates filament–membrane coupling under geometric confinement remains unclear. Here we show that spontaneous curvature fundamentally reorganizes filament–membrane morphogenesis in vesicles by biasing the competition between membrane deformation and filament bending. Using a unified curvature-elasticity framework that couples membrane and filament curvature energetics, we construct phase diagrams for vesicles enclosing filament loops and demonstrate that spontaneous curvature selects distinct symmetry-breaking pathways, leading to both continuous and discontinuous morphological transitions. In the rigid-filament limit, we further identify an intrinsic accommodation limit imposed by membrane elasticity together with area and volume conservation, beyond which no mechanically equilibrated configuration exists. These findings establish spontaneous curvature as a powerful design parameter for programming filament–membrane morphogenesis in biological and synthetic systems.

## Graphical abstract

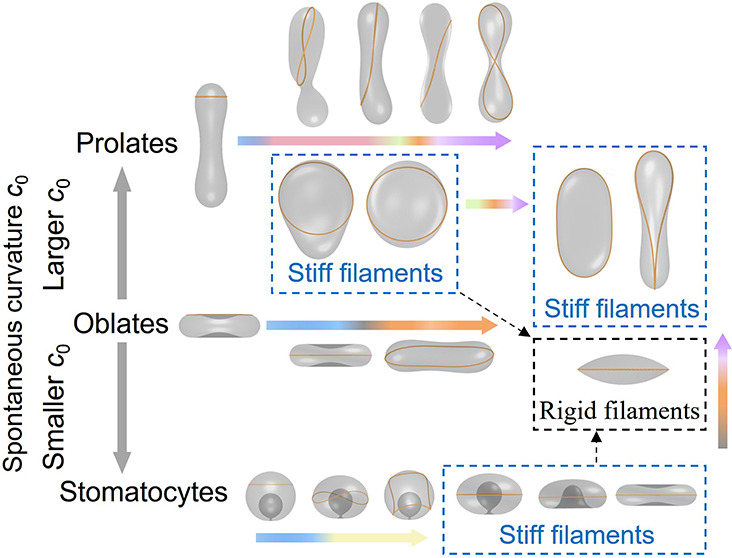

Lipid vesicles provide an ideal model system for studying how geometry and curvature elasticity govern shape transformations in soft matter and biological systems, with direct relevance to cellular processes such as endocytosis, organelle shaping, and synthetic-cell design.^1^ Vesicle morphogenesis is primarily controlled by membrane spontaneous curvature, which biases local bending, and geometric confinement, commonly quantified by the reduced volume under fixed area and volume constraints.^2^ Spontaneous curvature arises from lipid compositional asymmetry, protein adsorption, or leaflet imbalance and drives preferred curvature states.^3,4^ Geometric confinement restricts admissible shapes and gives rise to canonical morphologies, including spherical, prolate, oblate, and stomatocyte-like shapes.^5^ Together, these effects govern a broad spectrum of membrane remodeling processes, from endocytosis, cargo wrapping, and budding of lipid droplets^5–12^ to global shape transformations of cells and vesicles^1,13,14^ and membrane adaptation to extreme biological environments.^15^ The interplay between spontaneous curvature and geometric confinement becomes substantially richer in the presence of internal filamentous structures.

In living cells and synthetic analogues, vesicles rarely exist as empty containers but instead encapsulate cytoskeletal filaments such as actin filaments and microtubules.^16,17^ In bottom-up synthetic-cell platforms, programmable cytoskeletal assemblies have been engineered to organize intracellular contents and mechanically reprogram compartment morphology,^18^ while reconstituted systems demonstrate that confinement can direct cytoskeletal organization.^19,20^ Vesicles containing confined filaments therefore provide a minimal model for filament–membrane coupling and exhibit a broad spectrum of morphologies.^21–28^ Depending on filament length, stiffness, and topology, open filaments generate cherry-, dumpling-, pebble-, and racket-like vesicle shapes,^22,26,29^ whereas closed loops yield planar and saddle-shaped filament configurations.^23,26,28^ Theoretical and numerical studies have further revealed filament buckling, vesicle stretching, and convex-to-concave transitions in filament–vesicle systems.^30,31^ Despite this progress, most studies have focused on elastic or active filaments confined by rigid boundaries^32–34^ or deformable vesicles interacting with effectively rigid fibers.^35^ However, how spontaneous curvature regulates filament–membrane morphogenesis through the coupled effects of filament elasticity, membrane deformability, and geometric confinement remains largely unexplored.

Here we present a theoretical investigation of a vesicle enclosing a closed filament loop, using a unified curvature-elasticity framework in which membrane bending follows the Helfrich description^36^ and filament mechanics is governed by the curvature of its centerline.^37^ We show that spontaneous curvature fundamentally reorganizes filament–membrane morphogenesis under geometric confinement. Closed filament loops are considered because topological closure imposes a nonlocal geometric constraint, enabling collective buckling and symmetry-breaking modes that are absent in boundary-dominated open filaments. Such looped configurations also capture the networked, quasi-closed organization of cytoskeletal filaments in cells. We map morphological phase behavior across filament length, stiffness, spontaneous curvature, and reduced volume. We show that spontaneous curvature selectively shifts the dominant elastic mode between membrane bending and filament deformation, thereby controlling the instability mechanisms that govern symmetry breaking. This curvature-dependent redistribution of elastic energy leads to distinct transition pathways and gives rise to both continuous and discontinuous morphological transitions. In the rigid-filament limit, we further identify an intrinsic accommodation threshold beyond which mechanically equilibrated configurations cease to exist.

Consider a filament–vesicle system (Figure 1) consisting of a closed, inextensible filament of total length *L* constrained to the inner surface of a lipid vesicle of fixed area *A*(= 4*πR* ^2^) and volume *V*, where *R* is the effective vesicle radius. The filament is modeled as a homogeneous wormlike chain with bending stiffness *D*. The vesicle membrane is characterized by bending rigidity κ and spontaneous curvature *c*_0_. The total system energy *E*_tot_, comprising the filament bending energy *E*_f_ and the membrane bending energy *E*_m_, is given by^36,37^

$$\begin{array}{c} E_{\mathrm{tot}}=E_{f}+E_{m},,E_{f}=\frac{D}{2}\int_{0}^{L}C^{2}ds,,E_{m}=\frac{\kappa }{2}\int {\left(2H+c_{0}\right)}^{2}dA \end{array}$$

where *C* and d*s* represent the local curvature and arclength element of the filament, respectively, while *H* and d*A* denote the mean curvature and surface element of the vesicle membrane, respectively. Here, *H* = −1/*R* for a spherical vesicle of radius *R* under the present sign convention. A positive *c*_0_ favors outward bending, whereas a negative *c*_0_ favors inward bending toward the vesicle interior. No torsional degree of freedom is included in the filament model.

**Figure 1:**
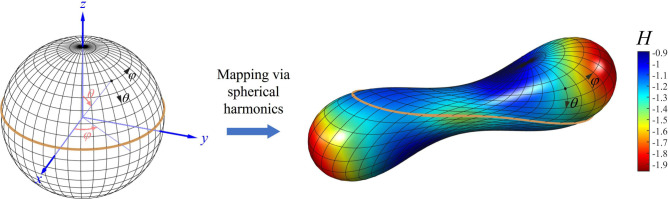
Schematic of the filament–vesicle system. A closed, inextensible filament loop (yellow) of length *L* is constrained on the inner surface of a vesicle with fixed area *A* and volume *V*. The vesicle surface is parametrized using spherical coordinates (θ, φ), mapping a reference sphere to the deformed shape. The local mean curvature of the membrane is denoted by *H*, which is negative for a spherical vesicle.

Rescaling energies by κ and lengths by *R*, the equilibrium configuration is governed by four dimensionless parameters: the stiffness ratio *D*/(*κR*), the normalized filament length *L*/*R*, the normalized spontaneous curvature *c*_0_ *R*, and the reduced volume *v* = 3*V*/(4*πR* ^3^). Fixing both membrane area and enclosed volume generally produces nonspherical vesicle shapes, thereby generating intrinsically anisotropic soft confinement for the filament. This confinement couples filament bending and reorientation to membrane deformation and underlies the morphology selection analyzed below.

To determine equilibrium configurations, we represent the vesicle shape using a spherical-harmonic parametrization^38^ and obtain stationary states via constrained minimization^39^ of *E*_tot_. Details of the parametrization and constrained minimization are provided in Note 1 of the Supporting Information (SI).

Before introducing the filament, we consider filament-free vesicles as reference states (Figure S1). We focus on two representative reduced volumes, *v* = 0.9 and 0.65, which define distinct confinement landscapes for the enclosed filament. At *v* = 0.9, the vesicle remains convex, whereas at *v* = 0.65, reduced volume induces concavity and necked confinement. These contrasting geometries provide complementary settings for probing filament-induced morphology selection.

The presence of filaments can significantly influence vesicle morphologies. To elucidate how filament–vesicle coupling governs morphology selection, we systematically vary the spontaneous curvature *c*_0_ *R* and the relative filament stiffness *D*/(*κR*) = 1, 10, and ∞ across *v* = 0.9 and 0.65.

We first consider convex confinement at *v* = 0.9. Representative equilibrium configurations for selected values of normalized filament length *L*/*R* and spontaneous curvature *c*_0_ *R*, at *D*/(*κR*) = 1 and 10, are shown in Figure 2. These configurations, classified using Schönflies point-group notation (Table S1), span seven symmetry classes. The corresponding morphological phase diagrams are given in Figure 3A for *D*/(*κR*) = 1 and Figure S2A for *D*/(*κR*) = 10. In the phase diagrams, dashed and solid boundaries denote continuous and discontinuous transitions, respectively, while the red boundary curves separate vesicle states with predominantly prolate-like and oblate-like geometric characters. These states retain the overall features of prolate or oblate vesicle shapes but allow filament-induced deviations.

**Figure 2:**
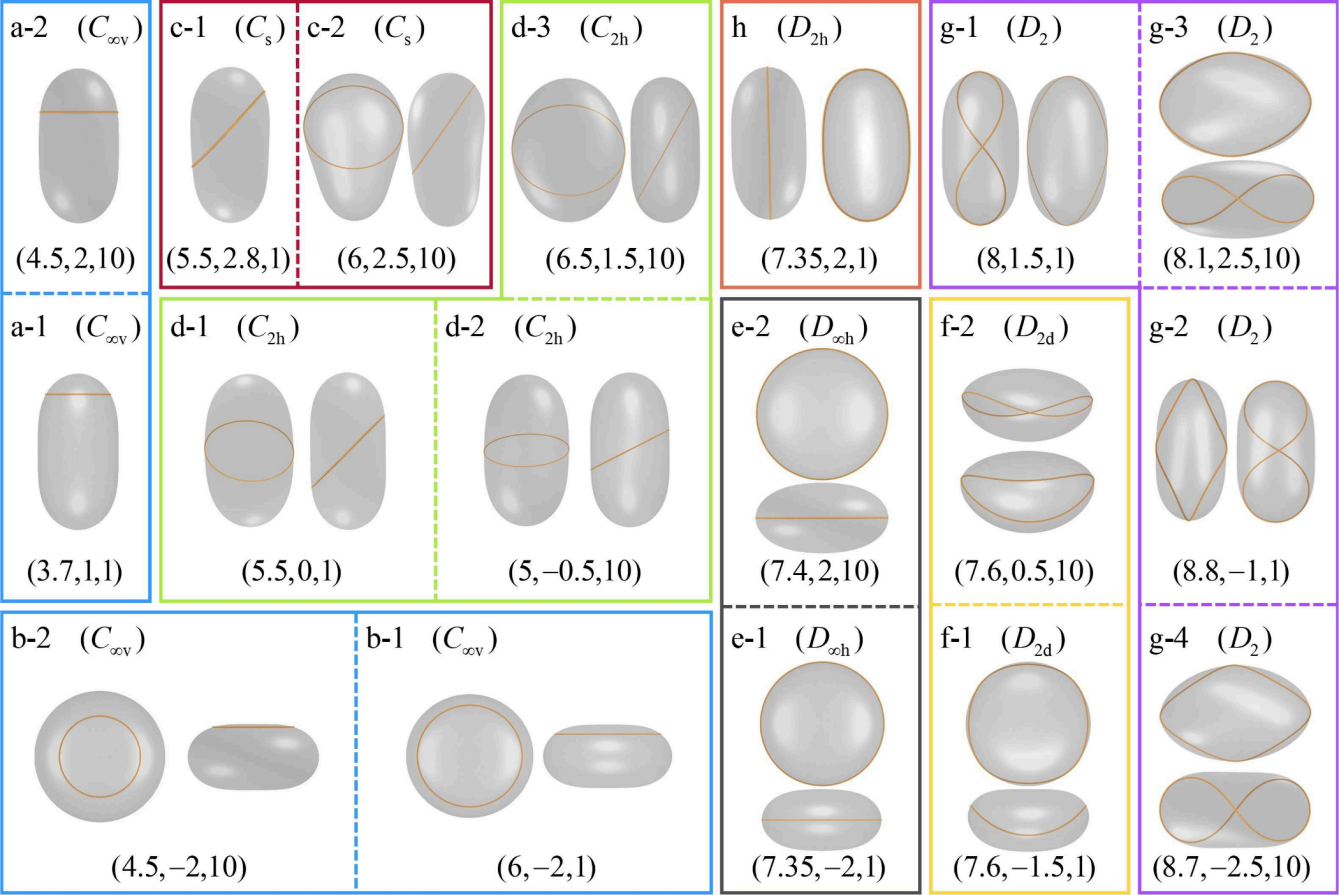
Morphologies of the filament–vesicle system at *v* = 0.9 for selected sets (*L*/*R*, *c*_0_ *R*, *D*/(*κR*)). Parameter values are indicated below each configuration. For clarity, selected configurations are shown from multiple viewing angles. Seven distinct symmetry classes are identified, with their corresponding point-group labels indicated in parentheses.

**Figure 3:**
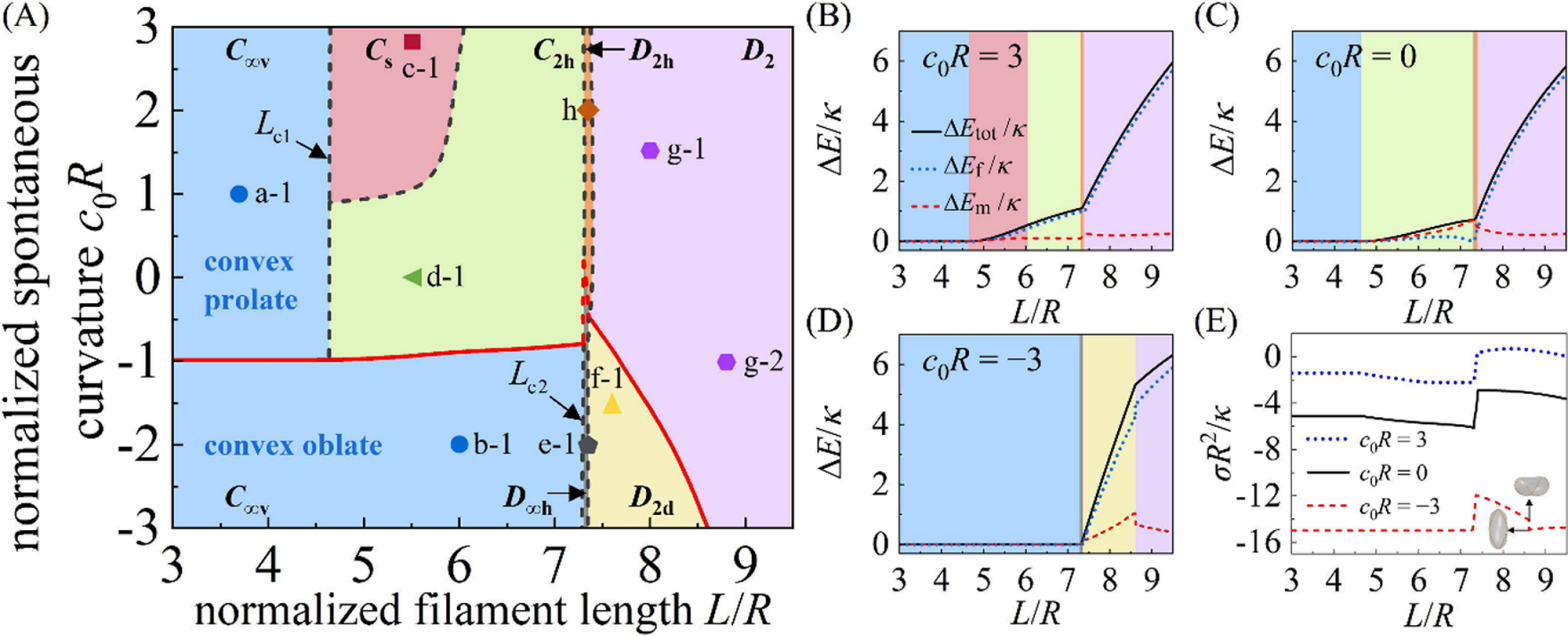
Curvature-dependent instability and phase behavior of the filament–vesicle system at *v* = 0.9 and *D*/(*κR*) = 1. (A) Morphological phase diagram in *L*/*R*–*c*_0_ *R* space, with symbols corresponding to the morphologies in Figure 2. Dashed and solid boundaries denote continuous and discontinuous transitions, respectively; the red boundary separates prolate- and oblate-derived vesicle states. (B–D) Profiles of the total energy change *ΔE*_tot_ and its components *ΔE*_f_ and *ΔE*_m_ at *c*_0_ *R* = 3, 0, and −3, respectively. (E) Profiles of the normalized membrane tension *σR* ^2^/κ for *c*_0_ *R* = −3, 0, and 3. Insets show representative configurations across the discontinuous transition. An enlarged view of the tension discontinuity and the corresponding energy profiles are provided in Figures S3 and S4. Positive (negative) σ corresponds to tensile (compressive) membrane tension.

For a filament-free vesicle (Figure S1A) at *v* = 0.9, the equilibrium shape is an axisymmetric convex prolate for *c*_0_ *R* > −0.984, with a maximum radial distance of 0.738*R* from the axis of revolution, and an axisymmetric convex oblate for *c*_0_ *R* < −0.984, with a maximum radial distance of 1.162*R*. In both cases, these characteristic radial distances are insensitive to *c*_0_ *R*, defining two thresholds for the coupled system. Accordingly, when *L* ≤ *L*_c1_ = 2π × 0.738*R* = 4.637*R* in the prolate vesicle state (e.g., Figure 2a-1,a-2), or *L* ≤ *L*_c2_ = 2π × 1.162*R* = 7.30*R* (e.g., Figure 2b-1,b-2) in the oblate vesicle state, the filament remains circular regardless of stiffness, and the system retains axisymmetric *C*_∞v_ symmetry (blue regions in Figures 3A and S2A). For *L* > *L*_c1_ or *L* > *L*_c2_, confinement-induced compression destabilizes the circular configuration, leading to filament reorientation and buckling and, consequently, successive symmetry breaking.

In the prolate regime, the post-threshold evolution depends strongly on *c*_0_ *R*. For relatively small |*c*_0_ *R*|, once *L* > *L*_c1_, confinement compresses the filament and drives symmetry breaking. Flexible filaments (*D*/(*κR*) = 1) deform readily, allowing the vesicle to remain prolate while the filament reorients into a nearly planar, oval-like configuration (e.g., Figure 2d-1). Stiff filaments (*D*/(*κR*) = 10) resist compression more strongly, maintaining a prolate vesicle at smaller *L* (e.g., Figure 2d-2) but inducing pronounced lateral distortion and eventually driving a transition to an oblate vesicle at larger *L* (e.g., Figure 2d-3). These *C*_2h_ configurations correspond to the green regions in Figures 3A and S2A. For sufficiently large positive *c*_0_ *R*, the vesicle becomes asymmetric, with one end expanding while the other contracts. The filament remains planar but relocates toward the more spacious end, yielding *C*_s_ system configurations that retain a single mirror plane while losing centrosymmetry (e.g., Figure 2c-1,c-2).

In the oblate regime, when *L* > *L*_c2_, the equatorial filament loop first expands radially under membrane compression while the coupled system remains axisymmetric with inversion symmetry (e.g., Figure 2e-1; *D*_∞h_). Flexible filaments sustain only limited radial expansion before buckling, whereas stiffer filaments slightly enlarge the *D*_∞h_ regime by more effectively resisting compression. With further increases in *L*, out-of-plane bending becomes favorable, and the filament loop evolves into a saddle-like configuration, leading to *D*_2d_ system symmetry (Figure 2f-1), followed at larger *L* by apparently twisted, figure-eight-like states (*D*_2_ system symmetry) (Figure 2g-2). Here, “twisted” refers only to the visual appearance of the filament configuration in certain viewing projections (Figure 2g-1 to 2g-4), rather than elastic torsion of the filament cross-section. Increased filament stiffness favors straighter segments, thereby promoting the *D*_2d_-to-*D*_2_ transition to reduce bending in the two half-loops (Figure 2f-2,g-4).

At large positive *c*_0_ *R*, further elongation leads to distinct large-*L* pathways depending on filament stiffness. For flexible filament loops, the oval loops narrow further and align within a plane, yielding *D*_2h_ symmetry (Figure 2h). A further increase in *L* triggers a supercritical symmetry-breaking instability that breaks reflection symmetry and drives a *D*_2h_-to-*D*_2_ transition (Figure 2g-1). This transition is identified as supercritical because the filament configuration evolves continuously from a planar oval loop to a spatial out-of-plane buckled state, with the out-of-plane deformation amplitude growing smoothly from zero and without a discontinuous jump in morphology or coexistence of metastable configurations, distinguishing this transition from first-order or subcritical instabilities.

In contrast, stiff filaments resist compression from the confining vesicle, leading instead to vesicle flattening while preserving vertical symmetry and maintaining a *D*_∞h_ symmetry (Figure 2e-2). Further increases in *L* lead to *D*_2d_ and *D*_2_ symmetry (Figure 2g-3).

The phase behavior is governed by a curvature-dependent redistribution of elastic energy between membrane bending and filament deformation. Comparing the phase diagrams for flexible and stiff filaments (Figures 3A and S2A) reveals that filament stiffness primarily restricts configurations involving strong filament narrowing, eliminating the *D*_2h_ region in the stiff-filament case.

To elucidate this mechanism, we analyze the energy decomposition for flexible filaments (*D*/(*κR*) = 1) in Figure 3B–D, where *ΔE*_tot_ = Δ*E*_m_ + Δ*E*_f_ is measured relative to a filament-free vesicle at *v* = 0.9 with varying *c*_0_ and a circular filament of length *L*. Accordingly, the membrane energy change is *ΔE*_m_ = *E*_m_ – *E*_m0_ and the filament bending energy change is *ΔE*_f_ = *E*_f_ – 2π^2^ *D*/*L*, where *E*_m0_ = 7.25κ, 29.90κ, and 164.18κ at *c*_0_ *R* = 3, 0, and −3, respectively.

For *c*_0_ *R* = 3 and 0, the system maintains its unperturbed ground state (*ΔE*_tot_ = 0) for *L* ≤ *L*_c1_, whereas for *c*_0_ *R* = −3, the corresponding threshold is *L*_c2_ = 7.30*R*. Beyond these thresholds, spontaneous curvature determines which elastic contribution dominates the deformation. At high positive curvature (c_0_ *R* = 3, Figure 3B), *ΔE*_f_ dominates throughout. At *c*_0_ *R* = 0, *ΔE*_m_ dominates at intermediate *L*/*R*, whereas *ΔE*_f_ regains dominance at large *L*/*R* (Figure 3C). In contrast, at *c*_0_ *R* = −3, deformation beyond *L*_c2_ is again primarily governed by *ΔE*_f_ (Figure 3D). A parallel energetic analysis for stiff filaments is presented in Figure S2B–D.

This curvature-dependent redistribution of elastic energy directly determines the instability modes that drive symmetry breaking and thus shapes the phase diagram in Figure 3A. Regions dominated by membrane bending favor gradual shape adaptation and continuous transitions, whereas filament-dominated regimes promote buckling-driven instabilities and are more likely to exhibit symmetry breaking.

The corresponding membrane-tension profiles in Figure 3E (*v* = 0.9 and *D*/(*κR*) = 1) provide independent signatures of these transitions. The membrane tension, σ­(= −∂*E*_tot_/∂A), is defined as a Lagrange multiplier conjugated to the fixed vesicle area *A*. For *c*_0_ *R* = 3 and 0, the sharp increase in σ marks continuous morphological transitions from *C*_2h_ to *D*_2h_ symmetry during vesicle flattening. For *c*_0_ *R* = −3, the initial sharp rise corresponds to a continuous *C*_∞v_-to-*D*_∞h_ transition, indicating membrane stretching as the equatorial filament loop expands radially. The subsequent abrupt drop in σ signifies a discontinuous *D*_2d_-to-*D*_2_ transition. The energy profiles underlying this discontinuous transition are shown in Figure S4, where the equilibrium boundary is determined by the crossing of two locally stable solution families. The overlap of locally stable solution families suggests possible path dependence under parameter sweeps.

For stiff filaments (*D*/(*κR*) = 10), σ shows a consistently sharp but continuous increase for *c*_0_ *R* = −3, 0, and 3 (Figure S5). This behavior accompanies continuous transitions to *D*_∞h_ symmetry (from *C*_2h_ for *c*_0_ *R* ≥ 0 and *C*_∞v_ for *c*_0_ *R* = −3), illustrating that increased filament stiffness amplifies the membrane’s tensile response during deformation.

Having established the filament–vesicle coupling mechanisms in the convex regime, we now turn to the more complex confinement landscape at *v* = 0.65, where reduced volume introduces pronounced concavity. The corresponding filament-free reference states (Figure S1B) exhibit three stable shapes separated by two critical spontaneous curvatures (*c*_0_ *R* = 0.006 and −0.3): concave prolate (dumbbell), concave oblate (discocyte), and stomatocyte shapes. Notably, the emergence of the noncentrosymmetric stomatocyte (*C*_∞v_) contrasts with the centrosymmetric (*D*_∞h_) shapes at *v* = 0.9, introducing lobe-selective confinement and a constricted neck. This geometric complexity provides a distinct confinement landscape for filament-induced morphogenesis.

These features lead to a substantially richer set of morphologies, as summarized in Figure 4 for flexible (*D*/(*κR*) = 1) and stiff (*D*/(*κR*) = 10) filaments. Eight symmetry classes are identified for flexible filaments, compared to six for stiff filaments, reflecting the additional deformation modes enabled by filament flexibility.

**Figure 4:**
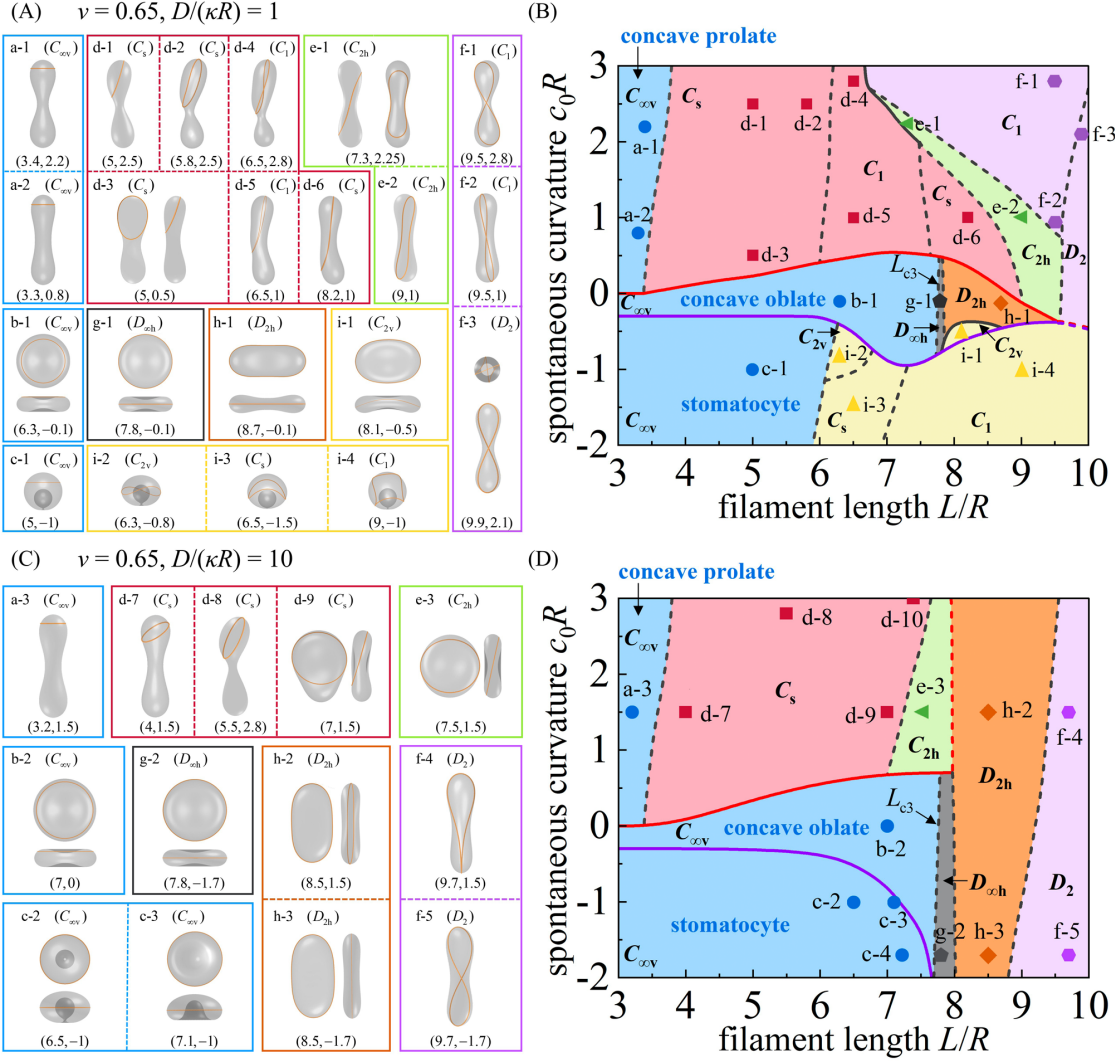
Morphologies and phase behavior of the filament–vesicle system at *v* = 0.65. (A,B) Flexible filaments, *D*/(*κR*) = 1. (A) Representative configurations for selected (*L*/*R*, *c*_0_ *R*); some configurations are shown from multiple viewing angles. Eight distinct symmetry classes are identified (labels in parentheses). (B) Corresponding phase diagram; symbols correspond to the representative configurations in (A). The threshold *L*_c3_ decreases slightly with decreasing *c*_0_ *R*, from *L*_c3_/*R* = 7.776 at *c*_0_ *R* = 0.485 to 7.734 at *c*_0_ *R* = −0.8. (C,D) Stiff filaments, *D*/(*κR*) = 10. (C) Representative configurations for selected (*L*/*R*, *c*_0_ *R*), showing six symmetry classes (labels in parentheses). (D) Corresponding phase diagram; symbols map to the configurations in (C). Configurations labeled c-4 and d-10 in (D) are shown in Figure S6. Here, *L*_c3_/*R* decreases from 7.76 at *c*_0_ *R* = 0.7 to 7.71 at *c*_0_ *R* = −2. In (B) and (D), dashed and solid lines indicate continuous and discontinuous morphological transitions, respectively. Red and purple boundaries separate prolate–oblate and oblate–stomatocyte vesicle states.

For *c*_0_ *R* > 0.006, the filament-free vesicle adopts an axisymmetric concave prolate shape with two enlarged end lobes. The corresponding maximum radial distance increases monotonically from 0.538*R* at *c*_0_ *R* = 0.006 to 0.604*R* at *c*_0_ *R* = 3, setting a curvature-dependent geometric threshold. Filament loops with radii below this scale remain circular, and the system retains axisymmetric *C*_∞v_ symmetry (Figure 4a-1 to 4a-3).

Beyond this threshold, two distinct deformation pathways emerge. At intermediate lengths, the filament remains confined within a single lobe, where confinement-induced compression drives reorientation and out-of-plane bending, producing *C*_s_ system configurations (Figure 4d-1 to 4d-3, 4d-7, and 4d-8), accompanied by lobe expansion. At larger *L*, the filament overcomes single-lobe confinement and penetrates the constricted neck, extending into the waist region and, in some cases, into the opposing lobe (Figure 4d-9 and Figure S6A for [Fig fig4]d-10). This transition introduces stronger geometric constraints and leads to lower-symmetry configurations, including *C*_1_, *C*_s_, *C*_2h_, and *D*_2_ states. The apparent twisting of these configurations further depends on the relative alignment between the filament and vesicle axes, with aligned axes observed in Figure 4f-3 and misaligned axes in Figure 4f-1 and 4f-2.

These observations indicate that, in contrast to the convex case, filament morphogenesis at *v* = 0.65 is governed not only by confinement-induced compression but also by the ability of the filament to overcome neck constraints and explore multilobe configurations.

In the range −0.3 < *c*_0_ *R* < 0.006, the filament-free vesicle adopts an axisymmetric concave oblate shape, with a maximum radial distance of approximately 1.234*R*. Filament loops shorter than the critical scale *L*_c3_ ≈ 2π × 1.234*R* = 7.753*R* remain circular, yielding axisymmetric *C*_∞v_ configurations. This threshold *L*_c3_ exhibits weak dependence on filament stiffness (Figures 4B,4D, and 5B).

**Figure 5:**
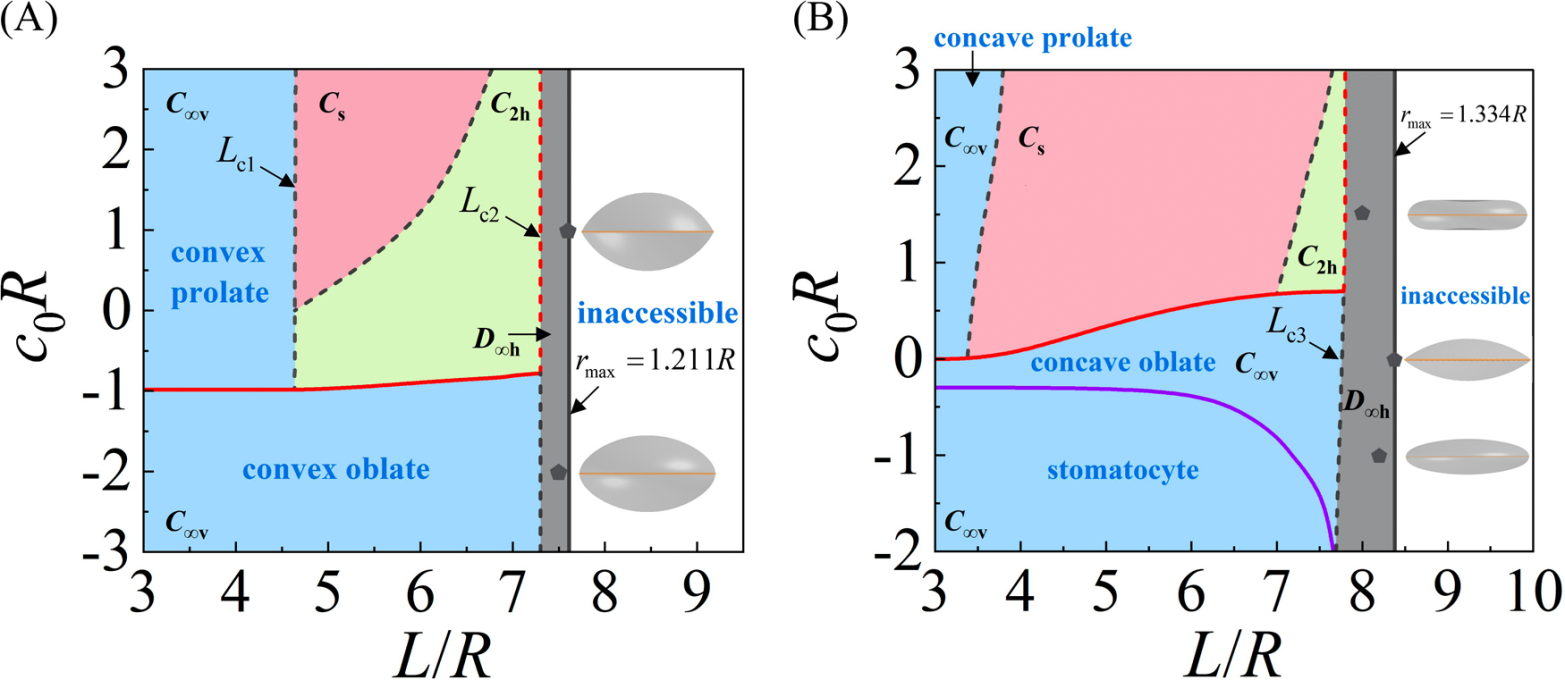
Morphological phase diagrams of the rigid filament–vesicle system at *v* = 0.9 (A) and 0.65 (B). Insets show representative equilibrium configurations marked by the pentagon symbols. Dashed and solid lines denote continuous and discontinuous morphological transitions, respectively. For *v* = 0.9, the critical filament lengths are *L*_c1_ = 4.637*R* and *L*_c2_ = 7.30*R*, with an upper bound *L*_max_ = 7.609*R*. For *v* = 0.65, the critical filament length *L*_c3_ decreases slightly with decreasing *c*_0_ *R*, from *L*_c3_/*R* = 7.8 at *c*_0_ *R* = 3 to 7.69 at *c*_0_ *R* = −2, and an upper bound *L*_max_ = 8.382*R*.

For *L* > *L*_c3_, the system first undergoes a radially symmetric expansion of the filament loop, accompanied by vesicle flattening and retaining *D*_∞h_ symmetry (Figure 4g-1,g-2). Upon further increase in *L*, the loop buckles either in-plane into an oval (*D*_2h_ system symmetry in Figure 4h-1 to 4h-3) or out-of-plane into a shallow saddle (*C*_2v_ symmetry in Figure 4i-1). The out-of-plane pathway is favored at lower *c*_0_ *R* but is suppressed for stiff filaments, explaining its absence in Figure 4D. For the flexible filaments, further reduction in *c*_0_ *R* stabilizes the stomatocyte geometry. Under this strongly concave confinement, the system undergoes a transition from *C*_2v_ to *C*_1_ symmetry, accompanied by pronounced out-of-plane deformation of the filament into highly distorted saddle-like shapes confined within the stomatocyte interior (Figure 4i-4).

For *c*_0_ *R* < −0.3, the filament-free vesicle adopts a stomatocyte shape. While short filaments remain circular (Figure 4c-1), the subsequent evolution becomes strongly stiffness-dependent. Flexible filaments (*D*/(*κR*) = 1) undergo buckling within the confined interior, forming saddle-like configurations with *C*_2v_ system symmetry for *c*_0_ *R* > −1.15 (Figure 4i-2), or *C*_s_ symmetry for *c*_0_ *R* < −1.15 (Figure 4i-3). Further elongation leads to strong out-of-plane deformation and reorientation, ultimately resulting in complete symmetry breaking (*C*_1_ symmetry in Figure 4i-4). In contrast, stiff filaments (*D*/(*κR*) = 10) resist buckling and instead deform the vesicle, leading to expansion and flattening of the stomatocyte (Figure 4c-2) and eventually widening of the constricted neck (Figure 4c-3 and Figure S6B for [Fig fig4]c-4).

The underlying physical mechanisms are analyzed in detail in the SI Note 2. Figures S7 and S8 show that these transitions are governed by curvature-dependent redistribution of elastic energy between membrane reshaping and filament bending, with membrane tension providing complementary signatures of both continuous and discontinuous transitions.

We finally consider the rigid-filament limit, which isolates the geometric constraints imposed by vesicle confinement. In this limit, the filament remains strictly circular and is restricted to rigid-body translation and rotation. As a result, symmetry classes that rely on in-plane or out-of-plane filament bending, such as *D*_2h_, *D*_2d_, and *D*_2_, are excluded (Figure 5). The accessible states are therefore limited to axisymmetric prolate, oblate, and stomatocyte shapes (*C*_∞v_), along with a small set of nonaxisymmetric states (*C*_s_ and *C*_2h_) arising from rigid-body rotation of the filament.

In contrast to flexible filaments, the rigid-filament limit introduces a strict upper bound on the admissible filament length, determined by the geometric capacity of the confining vesicle. For *v* = 0.9, the axisymmetric vesicle possesses a maximum equatorial radius *r*_max_ = 1.211*R*, essentially independent of *c*_0_ *R*. This sets an upper bound on the filament length *L*_max_ = 2*πr*_max_ = 7.609*R* (Figure 5A). For *v* = 0.65, the vesicle has a larger *r*_max_ = 1.334*R*, leading to a correspondingly larger upper bound *L*_max_ = 8.382*R* (Figure 5B). Further numerical results show that *r*_max_/*R* as a function of *v* can be accurately approximated by the two-parameter expression *v* = {1 – [(*r*_max_/*R*)^2^ – 1]^*a*^}^*b*^, with fitted coefficients *a* = 2.22 and *b* = 0.505. Remarkably, the simpler expression

$$v=\sqrt{1-{\left[{\left(\frac{r_{\max}}{R}\right)}^{2}-1\right]}^{11/5}}$$

achieves nearly the same accuracy throughout the entire range *v* ∈ [0, 1].

The inaccessible region reflects the finite accommodation capacity of a vesicle with prescribed area and volume. Beyond a critical filament-imposed span, no mechanically equilibrated configuration can satisfy the geometric and confinement constraints. Similar inaccessible regimes reported for vesicles enclosing rigid straight rods^40^ suggest that this limit is not specific to loop geometry.

A central outcome of this study is that filament–vesicle morphologies are jointly controlled by reduced volume, membrane spontaneous curvature, and filament deformability through their effects on confinement geometry and elastic-energy redistribution. Reduced volume sets the global anisotropy of confinement: larger *v* favors weakly anisotropic, higher-symmetry states, whereas smaller *v* creates a pronounced waist and partitions the vesicle into lobes. Stiff filament loops tend to remain within one lobe and expand it, while flexible loops can traverse the constricted neck, at the cost of both filament bending and deformation of highly curved membrane regions. Spontaneous curvature further biases these pathways by stabilizing compatible membrane shapes. This contrasts with nearly spherical vesicles, where isotropic confinement does not favor specific buckling directions and filament loops mainly retain higher-symmetry configurations.^26^ The comparison with rigid filament limits highlights the role of deformability: elastic filaments adapt to anisotropic confinement and stabilize low-symmetry states, whereas rigid filaments are restricted by geometric bounds.

This mechanical picture provides a direct bridge to membrane-confined closed or quasi-closed cytoskeletal structures. Ring-like filament assemblies have been observed in reconstituted vesicle systems, where polymerizing actin can self-organize into equatorial rings and cross-linked actin can form stable bundles inside giant vesicles.^21,24^ A close cellular example is the platelet marginal band, a peripheral microtubule ring that supports the discoid platelet shape and buckles into saddle-like configurations during activation as the cell rounds.^23,41^ Because such loops lack free ends, they cannot relax confinement by end displacement and instead undergo global reorientation, in-plane narrowing, out-of-plane buckling, and symmetry breaking, consistent with the circular, oval, saddle-like, and figure-eight-like configurations predicted here.

The relevance also extends to membrane remodeling and synthetic-cell design. Dynamin and ESCRT-III remodel membranes through filament reorganization, neck constriction, and curvature-driven shape changes.^42^ Beyond single-vesicle morphogenesis, flattened oblate or discotic vesicles can serve as geometrically compatible units for stacked or rouleaux-like assemblies,^43^ reminiscent of layered Golgi cisternae used for compartmentalized processing.^44^ Figure 4 shows that filament length, stiffness, and spontaneous curvature regulate transitions among oblate-like, stomatocyte-like, and asymmetric states, suggesting a passive route to program flattened, invaginated, or asymmetric vesicle geometries. Although the present model does not simulate division, recent lipid-nucleotide multilamellar protocells show that caveola formation, core–shell asymmetry, and shell reconfiguration can drive asymmetric splitting into morphologically distinct progeny,^45^ further supporting the relevance of curvature- and confinement-mediated remodeling.

## Supplementary Material





## Notes

MATLAB .fig files for representative filament–vesicle configurations (ZIP)
Details of the spherical-harmonic parametrization, constrained energy minimization, symmetry classification, and supplementary figures of representative configurations, phase diagrams, energy decomposition, and membrane tension (PDF)
