# Supplementary material for: Spontaneous Curvature Sculpts Filament–Vesicle Morphogenesis
Source: Nano Lett. 2026 Jul 23;26(31):10273–80. doi: 10.1021/acs.nanolett.6c02160 (PMC13475500; doi:10.1021/acs.nanolett.6c02160)
Supplement: Supplementary file 1 [file nl6c02160_si_002.pdf]

Supporting Information for

Spontaneous curvature sculpts filament–vesicle morphogenesis

Yaxin Fang, Chengyao Zhang, Meng Wang, Chao Shi, Xin Yi*

School of Mechanics and Engineering Science, Peking University, Beijing, China

*E-mail: xyi@pku.edu.cn

This PDF file includes:

Supporting text

Figures S1 to S8

Tables S1 and S2

Other Supporting Information files:

MATLAB .fig files for representative filament–vesicle configurations

Note 1. Representation of the filament–vesicle system and its energy minimization

The vesicle surface is represented using a spherical harmonic parameterization. In a Cartesian coordinate system, the position vector $\mathbf{r}^{\mathrm{m}}$ of the vesicle surface is represented as (ref. 38)

$$\mathbf{r}^{\mathrm{m}}(\theta,\varphi)=\begin{bmatrix} x^{\mathrm{m}}(\theta,\varphi) \\ y^{\mathrm{m}}(\theta,\varphi) \\ z^{\mathrm{m}}(\theta,\varphi) \end{bmatrix}=\begin{bmatrix} \sum_{l=0}^{l_{\max}}\sum_{k=-l}^{l} C_{lk}^{x} Y_{lk}(\theta,\varphi) \\ \sum_{l=0}^{l_{\max}}\sum_{k=-l}^{l} C_{lk}^{y} Y_{lk}(\theta,\varphi) \\ \sum_{l=0}^{l_{\max}}\sum_{k=-l}^{l} C_{lk}^{z} Y_{lk}(\theta,\varphi) \end{bmatrix},$$

where $\theta\in[0,\pi]$ and $\varphi\in[0,2\pi]$ denote the polar and azimuthal angles in a spherical coordinate system, respectively (Figure 1), and $C_{lk}^{x,y,z}$ are control coefficients. In our calculations, the spherical harmonic expansions are truncated at $l = l_{\max}$. Since each Cartesian coordinate contains $(l_{\max} + 1)^2$ real spherical harmonic modes, the vesicle shape is described by $3(l_{\max} + 1)^2$ control coefficients. We use $l_{\max} = 5$ for prolate and oblate vesicle shapes and $l_{\max} = 7$ for stomatocyte shapes, for which additional modes are required to resolve the invaginated neck region. Convergence tests (not shown here) performed for representative nonspherical equilibrium states confirm that these truncation orders are sufficient to capture the equilibrium shapes and energy components reported in this work.

Here, $Y_{lk}(\theta,\varphi)$ is the real spherical harmonic function defined as

$$Y_{lk}(\theta,\varphi)=\begin{cases} \sqrt{2}N_{lk}P_{lk}(\cos\theta)\cos(k\varphi), & k>0, \\ N_{l0}P_{l0}(\cos\theta), & k=0, \\ \sqrt{2}N_{lk}P_{lk}(\cos\theta)\sin(-k\varphi), & k<0, \end{cases}$$

with

$$N_{lk}=\sqrt{\frac{(2l+1)(l-|k|)!}{4\pi(l+|k|)!}} \quad \text{and} \quad P_{lk}(x)=(-1)^{|k|}\frac{1-x^2}{2^l l!}\frac{\mathrm{d}^{l+|k|}[(x^2-1)^l]}{\mathrm{d}x^{l+|k|}}.$$

For the surface-constrained filament, the filament centerline is prescribed as a curve on the vesicle surface. Its position vector $\mathbf{r}^{\mathrm{f}}$ is written as $\mathbf{r}^{\mathrm{f}}(\varphi) = \mathbf{r}^{\mathrm{m}}(\theta_0,\varphi)$ with θ_0 as a prescribed polar angle. Therefore, the filament is not independently parameterized; its geometry is determined by the same spherical harmonic coefficients $C_{lk}^{x,y,z}$ that define the vesicle surface. The geometrical quantities of both the vesicle surface and the filament, including surface curvatures and filament curvature, are computed from $\mathbf{r}^{\mathrm{m}}(\theta,\varphi)$ and $\mathbf{r}^{\mathrm{f}}(\varphi)$ using classical differential geometry.

For the vesicle surface represented by $\mathbf{r}^{\mathrm{m}}(\theta,\varphi)$, its local mean curvature H is given by

$$H=\frac{EN-2FM+GL}{2(EG-F^2)},$$

where $E=\mathbf{r}^{\mathrm{m}}_{,\theta}\cdot\mathbf{r}^{\mathrm{m}}_{,\theta}$, $F=\mathbf{r}^{\mathrm{m}}_{,\theta}\cdot\mathbf{r}^{\mathrm{m}}_{,\varphi}$, $G=\mathbf{r}^{\mathrm{m}}_{,\varphi}\cdot\mathbf{r}^{\mathrm{m}}_{,\varphi}$, $L=\mathbf{r}^{\mathrm{m}}_{,\theta\theta}\cdot\mathbf{n}^{\mathrm{m}}$, $M=\mathbf{r}^{\mathrm{m}}_{,\theta\varphi}\cdot\mathbf{n}^{\mathrm{m}}$, and $N=\mathbf{r}^{\mathrm{m}}_{,\varphi\varphi}\cdot\mathbf{n}^{\mathrm{m}}$ are coefficients of the first and second fundamental forms of the surface at the point $\mathbf{r}^{\mathrm{m}}$, with $\mathbf{n}^{\mathrm{m}}=(\mathbf{r}^{\mathrm{m}}_{,\theta}\times\mathbf{r}^{\mathrm{m}}_{,\varphi})/|\mathbf{r}^{\mathrm{m}}_{,\theta}\times\mathbf{r}^{\mathrm{m}}_{,\varphi}|$ as the outward unit normal vector to the surface. H is negative for a spherical vesicle. Henceforth, a subscript comma followed by a variable indicates partial differentiation with respect to that variable (e.g., $\mathbf{r}^{\mathrm{m}}_{,\theta}\equiv\partial\mathbf{r}^{\mathrm{m}}/\partial\theta$).

The area element of the vesicle surface is $\mathrm{d}A = |\mathbf{r}^{\mathrm{m}}_{,\theta} \times \mathbf{r}^{\mathrm{m}}_{,\varphi}|\,\mathrm{d}\theta\mathrm{d}\varphi = \sqrt{g}\,\mathrm{d}\theta\mathrm{d}\varphi$, and the surface area is $A = \int_0^{2\pi}\int_0^{\pi}\sqrt{g}\,\mathrm{d}\theta\mathrm{d}\varphi$.

The vesicle volume is $V = \frac{1}{3}\int_0^{2\pi}\int_0^{\pi}\mathbf{r}^{\mathrm{m}}\cdot(\mathbf{r}^{\mathrm{m}}_{,\theta}\times\mathbf{r}^{\mathrm{m}}_{,\varphi})\mathrm{d}\theta\mathrm{d}\varphi$.

The bending energy of the vesicle membrane is $E_{\mathrm{m}} = \frac{\kappa}{2}\int_0^{2\pi}\int_0^{\pi}(2H + c_0)^2\sqrt{g}\,\mathrm{d}\theta\mathrm{d}\varphi$.

For the filament represented by $\mathbf{r}^{\mathrm{f}}(\varphi)$, its local curvature is $C = \frac{|\mathbf{r}^{\mathrm{f}}_{,\varphi}\times\mathbf{r}^{\mathrm{f}}_{,\varphi\varphi}|}{|\mathbf{r}^{\mathrm{f}}_{,\varphi}|^3}$ and its bending energy is $E_{\mathrm{f}} = \frac{D}{2}\int_0^{2\pi}\frac{|\mathbf{r}^{\mathrm{f}}_{,\varphi}\times\mathbf{r}^{\mathrm{f}}_{,\varphi\varphi}|^2}{|\mathbf{r}^{\mathrm{f}}_{,\varphi}|^5}\mathrm{d}\varphi$.

The vesicle area A, the enclosed volume specified by the reduced volume v, and the filament length L are imposed as equality constraints in the numerical minimization. The total elastic energy E_{tot}, expressed as a function of the spherical harmonic control coefficients $C_{lk}^{x,y,z}$, is minimized using the MATLAB fmincon routine with the interior-point algorithm for constrained nonlinear optimization. The optimization variables are the spherical harmonic coefficients $C_{lk}^{x,y,z}$ defining the vesicle surface. Since the filament centerline is constrained to lie on the vesicle surface, its configuration is also determined by $C_{lk}^{x,y,z}$.

Using the interior-point method (ref. 39), both first- and second-order derivatives of the total energy and equality constraints with respect to $C_{lk}^{x,y,z}$ are supplied either explicitly or implicitly. The interior-point algorithm then iteratively solves the constrained minimization problem by satisfying the corresponding Karush–Kuhn–Tucker (KKT) optimality conditions. First-order derivatives (gradients) determine descent directions and KKT optimality criteria. Second-order derivatives (Hessian matrices) capture local curvature of objective functions. They enable quadratic convergence of Newton iterations, greatly accelerating the interior-point method. Quasi-Newton techniques approximate Hessian matrices using gradient data to reduce computational cost. Upon convergence, the optimized coefficients determine the equilibrium vesicle shape, filament configuration, and corresponding energy components.

For each prescribed set of model parameters, including v, c_0R, L/R, and $D/(\kappa R)$, multiple optimization trials are performed from different initial configurations. These include neighboring solutions obtained by continuation in parameter space, simple reference shapes, and symmetry-perturbed configurations. The converged stationary configurations are compared by their total elastic energies, and the lowest-energy configuration is selected as the equilibrium state for constructing the morphological phase diagrams. Discontinuous boundaries are identified from energy crossings between distinct low-energy solution families, whereas continuous transitions are determined from smooth variations in morphology, symmetry, total energy, and membrane tension.

Note 2. Supplementary numerical results

A. Symmetry classifications in Schönflies notation

In this work, equilibrium configurations of the filament–vesicle system are classified according to their symmetry elements using Schönflies notation (Table S1). The distinct symmetry point groups identified in the phase diagrams are defined based on the presence of rotation axes, reflection planes, and inversion centers.

Table S1. The Schönflies point groups used in this work.

Point groups	Definitions
$C_{\infty v}$	axisymmetric configurations with a principal axis of infinite-fold rotational symmetry and infinitely many vertical mirror planes, but without a horizontal mirror plane or inversion center
$D_{\infty h}$	axisymmetric configurations with a principal axis of infinite-fold rotational symmetry, infinitely many vertical mirror planes, a horizontal mirror plane, and an inversion center
C_s	configurations possessing a single mirror plane
C_{2h}	configurations possessing a two-fold rotation axis, a horizontal mirror plane, and an inversion center
D_{2d}	configurations with a four-fold improper rotation axis, two mutually perpendicular two-fold axes, and two vertical dihedral mirror planes
D_2	configurations with three mutually perpendicular two-fold rotation axes and no mirror plane
D_{2h}	configurations with three mutually perpendicular two-fold rotation axes, three mutually perpendicular mirror planes, and an inversion center
C_{2v}	configurations with a two-fold rotation axis and two mutually perpendicular vertical mirror planes intersecting at the axis
C_1	configurations with no symmetry other than the identity

B. Equilibrium morphologies of filament-free vesicles

We characterize the equilibrium morphologies of filament-free vesicles as functions of the reduced volume v and normalized spontaneous curvature c_0R in Figure S1. At zero spontaneous curvature ($c_0 = 0$), the vesicle remains spherical at $v = 1$, and transitions through prolate ($v \in (0.651,1)$), oblate ($v \in (0.591,0.651)$), and stomatocyte ($v \in (0,0.591)$) morphologies as v decreases.

When spontaneous curvature is introduced, the filament-free vesicle morphologies at given v become sensitive to c_0R. For a filament-free vesicle of $v = 0.9$, the vesicle adopts a convex prolate shape when $c_0R > -0.984$ and a convex oblate shape when $c_0R < -0.984$ (Figure S1A). At $v = 0.65$, the filament-free vesicle exhibits three stable morphologies depending on the value of c_0R: a concave prolate (dumbbell) shape ($c_0R > 0.006$), a concave oblate (discocyte) shape ($-0.3 < c_0R \leq 0.006$), and a stomatocyte ($c_0R \leq -0.3$) (Figure S1B).

All these vesicle morphologies are axisymmetric (Figure S1). The prolate and oblate branches belong to the $D_{\infty h}$ point group, whereas the stomatocyte shape is classified as $C_{\infty v}$ group (Table S1).

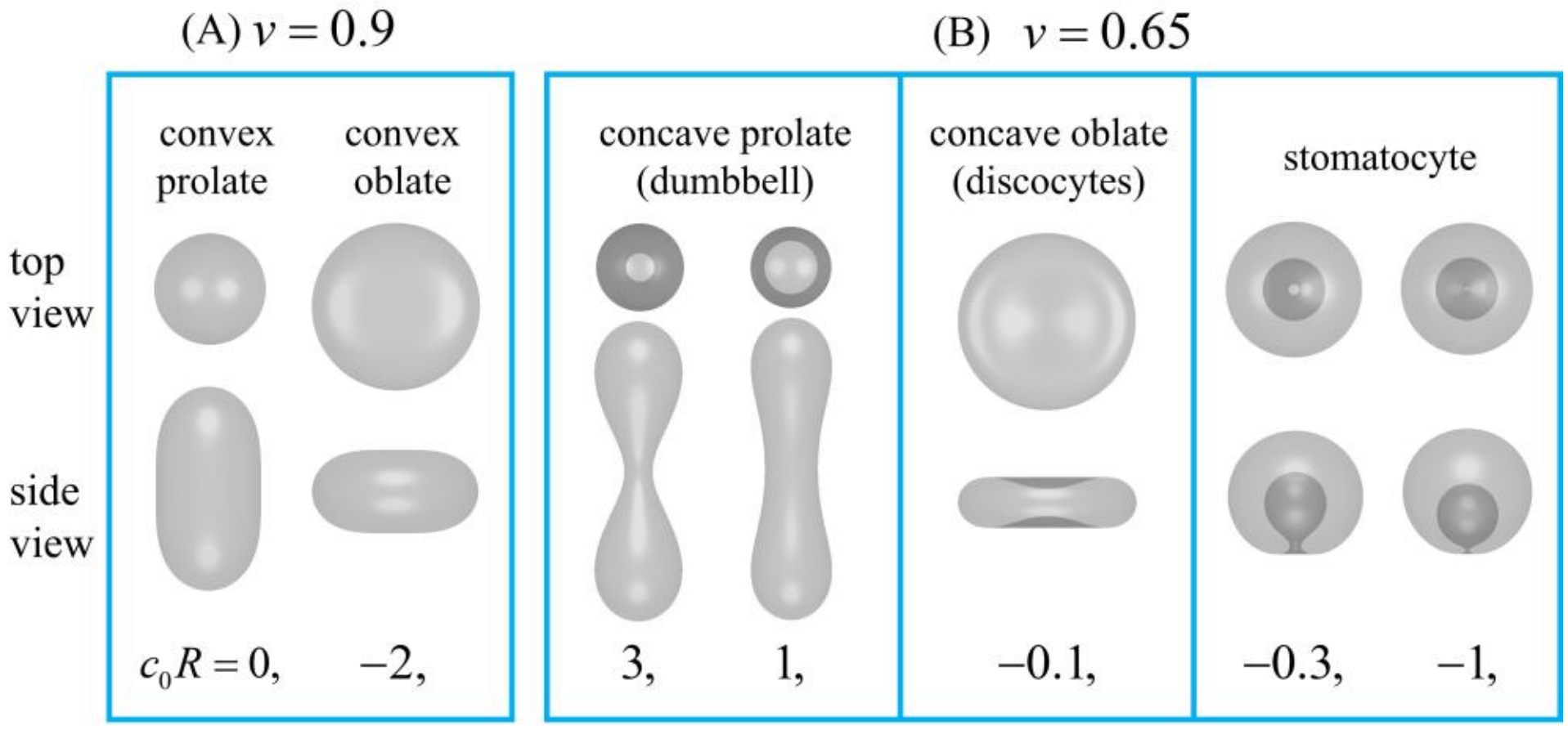

Figure S1. Selected axisymmetric vesicle morphologies at reduced volumes v = 0.9 (A) and 0.65 (B). Corresponding values of c_0R are indicated below each morphology. Our morphological predictions, obtained via spherical harmonic parameterization and numerical optimization, agree well with the axisymmetric solutions reported in ref. 2, which are derived by solving the Euler–Lagrange equations.

These filament-free equilibrium states define the soft-confinement geometries. In particular, the contrast between v = 0.9 and v = 0.65 is central to the morphology selection of the coupled filament–vesicle system: the former provides convex confinement and primarily probes filament-induced symmetry breaking, whereas the latter introduces lobe-selective and neck-mediated confinement, enabling qualitatively new transition pathways and lower-symmetry configurations.

C. Morphological phase diagrams and profiles of energy and membrane-tension at different reduced volumes

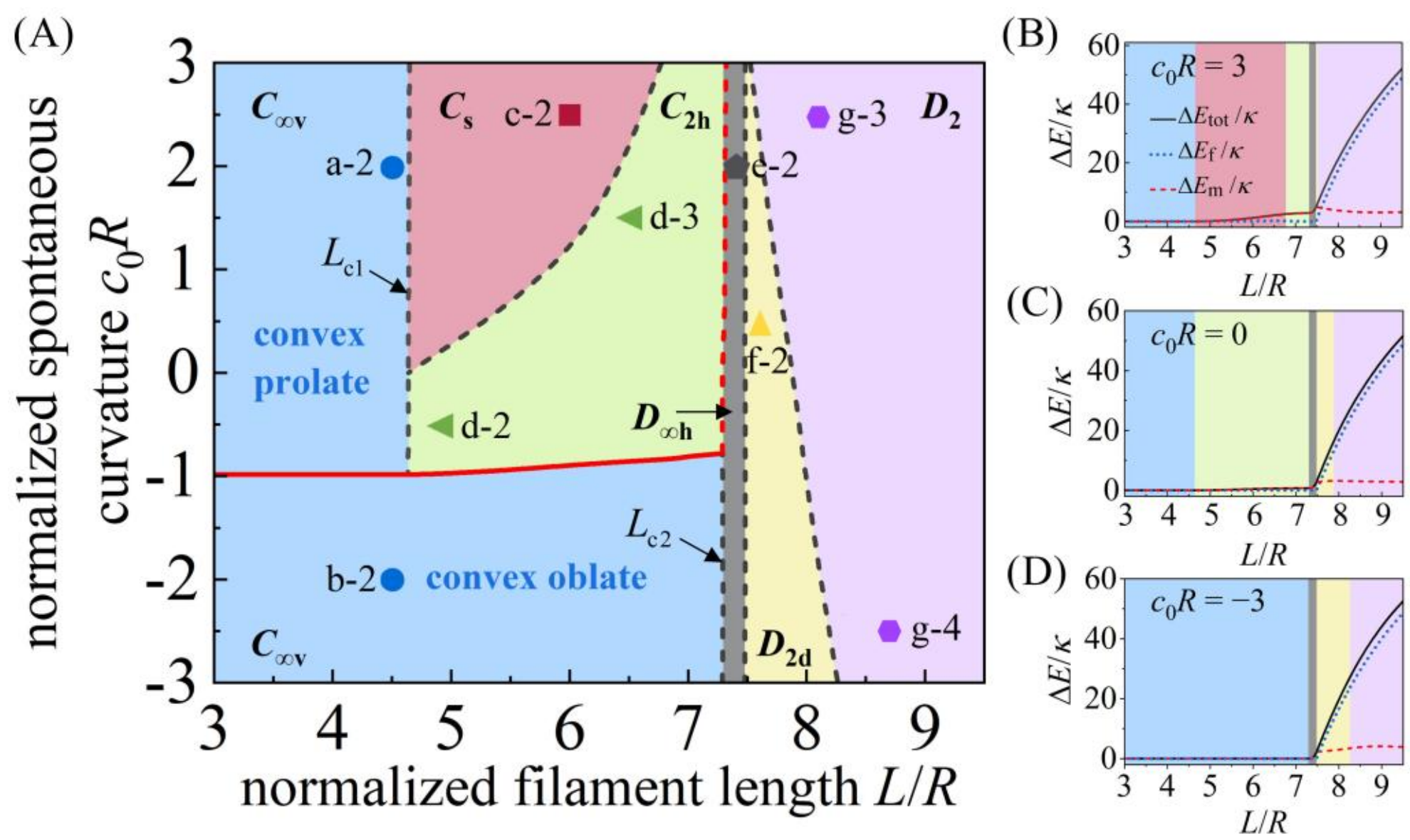

Figure S2. Morphological transitions and energy decomposition profiles at v = 0.9 and $D/(\kappa R)$ = 10. (A) Morphological phase diagram of the filament–vesicle system in L/R–c_0R space. Symbols correspond to the morphologies shown in Figure 2 in the main text. (B–D) Profiles of the total elastic energy change ΔE_{tot} and its components ΔE_f and ΔE_m at c_0R = 3, 0, and −3, respectively. Dashed (solid) phase boundaries indicate continuous (discontinuous) morphological transitions.

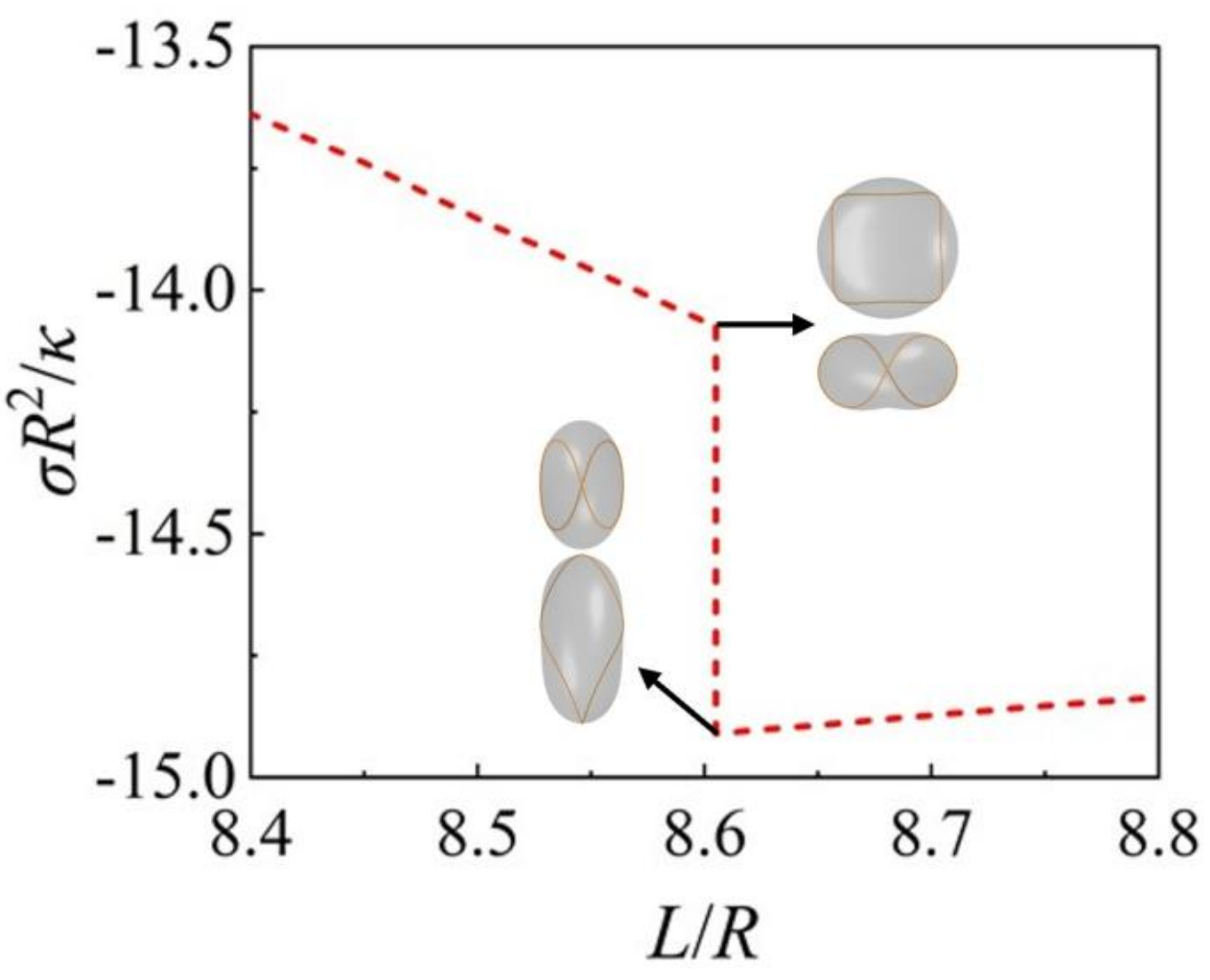

Figure S3. Enlarged view of the abrupt drop in the normalized membrane tension $\sigma R^2/\kappa$ at v = 0.9, $D/(\kappa R)$ = 1, and c_0R = −3, corresponding to the dotted red curve in Figure 3E. The two sets of configurations shown at L/R = 8.605 illustrate the transition from an oblate vesicle with a saddle-like filament to a prolate vesicle with a figure-eight-like filament. In each set, the upper image shows the top view and the lower image shows the side view.

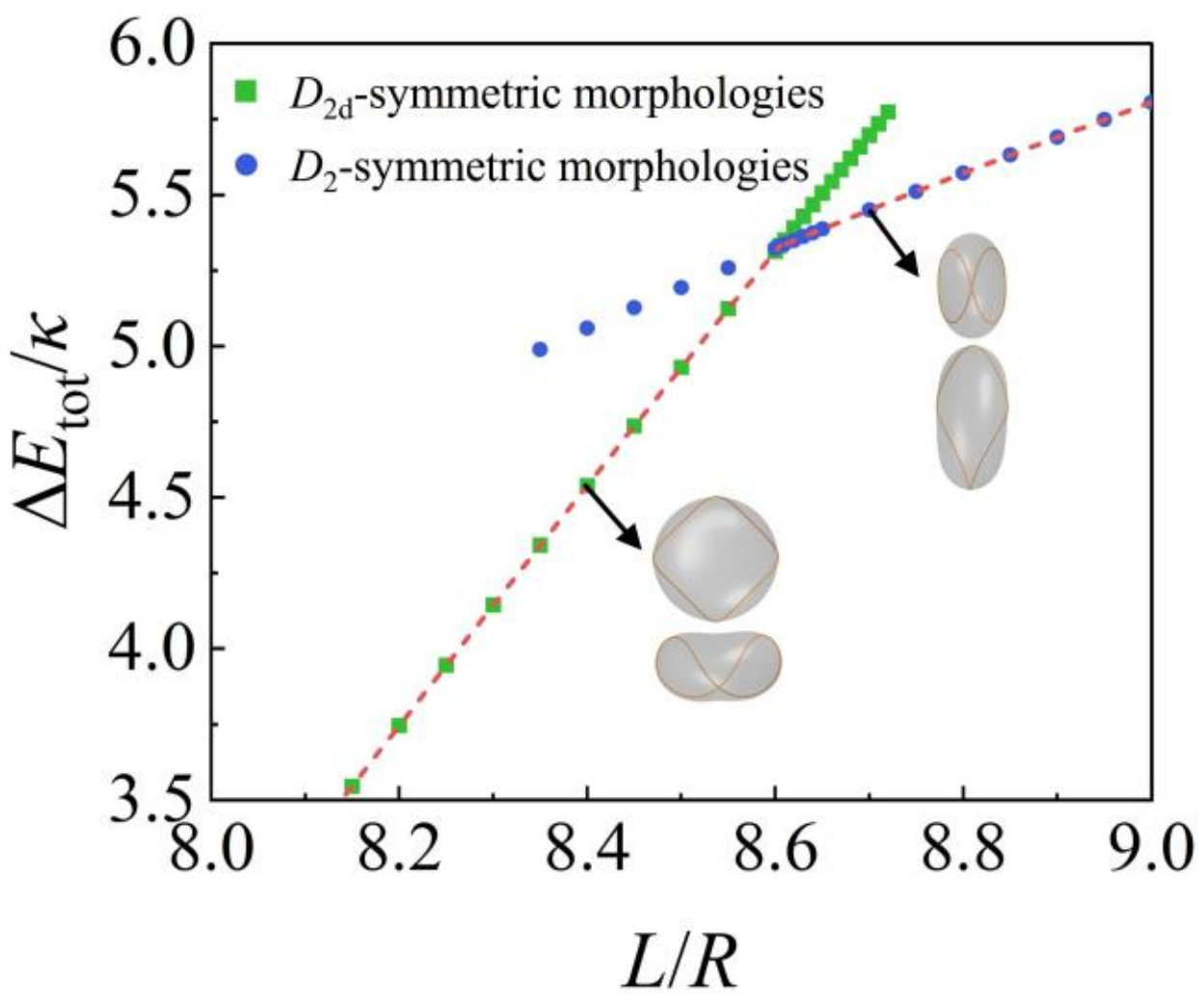

Figure S4. Total energy changes of the two locally stable morphological families across the D_{2d}-to-D_2 discontinuous transition boundary in Figure 3A at v = 0.9, $D/(\kappa R)$ = 1, and c_0R = −3. Green squares denote the D_{2d}-symmetric morphologies, and blue circles denote the D_2-symmetric morphologies. The dashed red segments represent the lower-energy envelope selected as the equilibrium energy. The energy crossing occurs at L/R = 8.605, where the equilibrium state switches discontinuously from the D_{2d}-symmetric morphology to the D_2-symmetric morphology. In each configuration set, the upper image shows the top view and the lower image shows the side view.

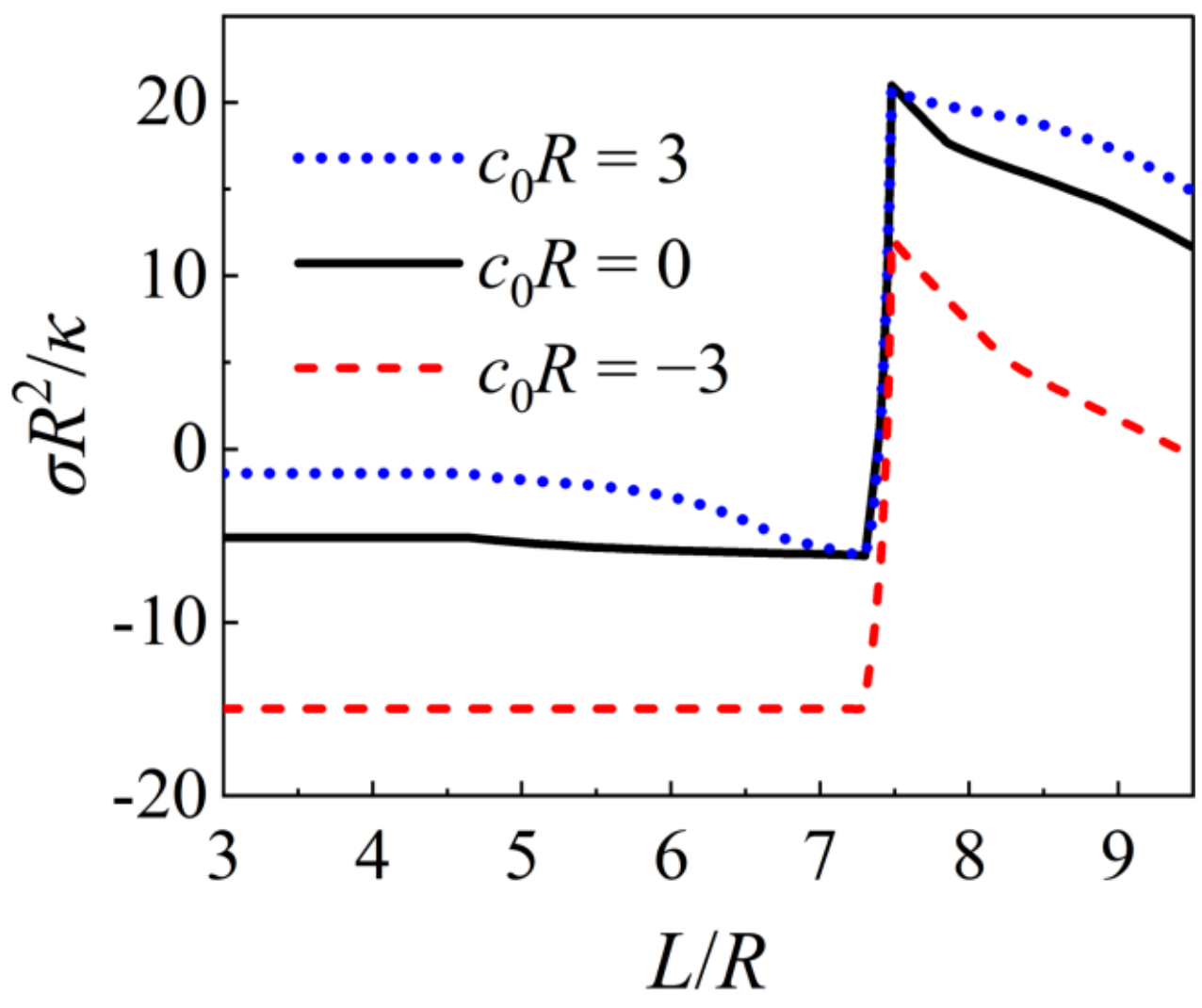

Figure S5. Profiles of the normalized membrane tension $\sigma R^2/\kappa$ at v = 0.9 and $D/(\kappa R)$ = 10 for c_0R = −3, 0, and 3. The morphological transitions here are continuous.

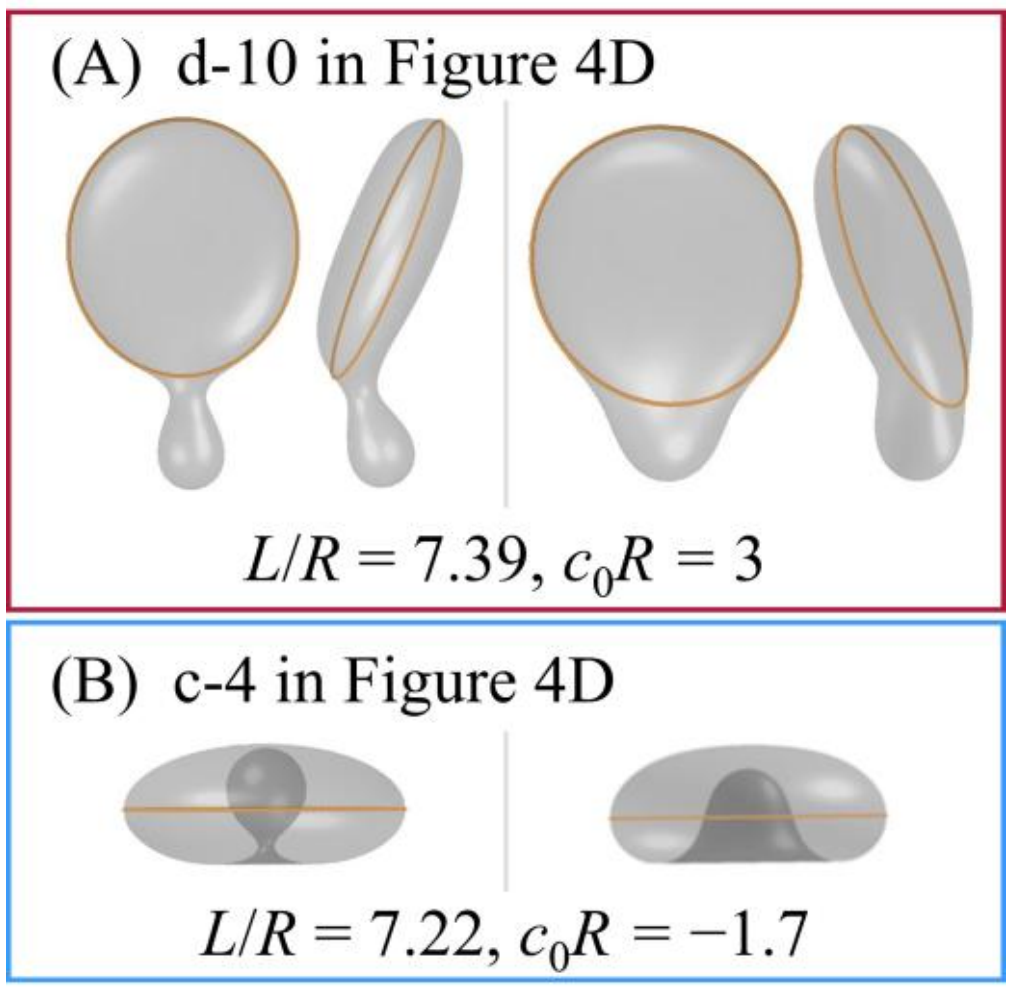

Figure S6. Selected system configurations at v = 0.65 and $D/(\kappa R)$ = 10. (A) c_0R = 3, L/R = 7.39; (B) c_0R = −1.7, L/R = 7.22. In each panel, the left and right images show two coexisting configurations at the same parameter values.

For the vesicles with reduced volume v = 0.65, stiff filaments of $D/(\kappa R)$ = 10 undergo negligible deformation, and the vesicle correspondingly conforms to the circular filament geometry. At c_0R = 3 and L/R = 7.39 (d-10 configurations in Figure 4D), two system configurations coexist: one retaining a narrow waist (left) and the other exhibiting a widened waist (right). The narrow-waist configuration becomes energetically unfavorable as L increases. Similarly, at c_0R = −1.7 and L/R = 7.22 (c-4 configurations in Figure 4D), stomatocyte vesicles display two different invaginated configurations: one with a narrow-necked invagination (left) and the other with a broad invagination without a pronounced neck (right). With increasing L, the latter configuration is energetically favored.

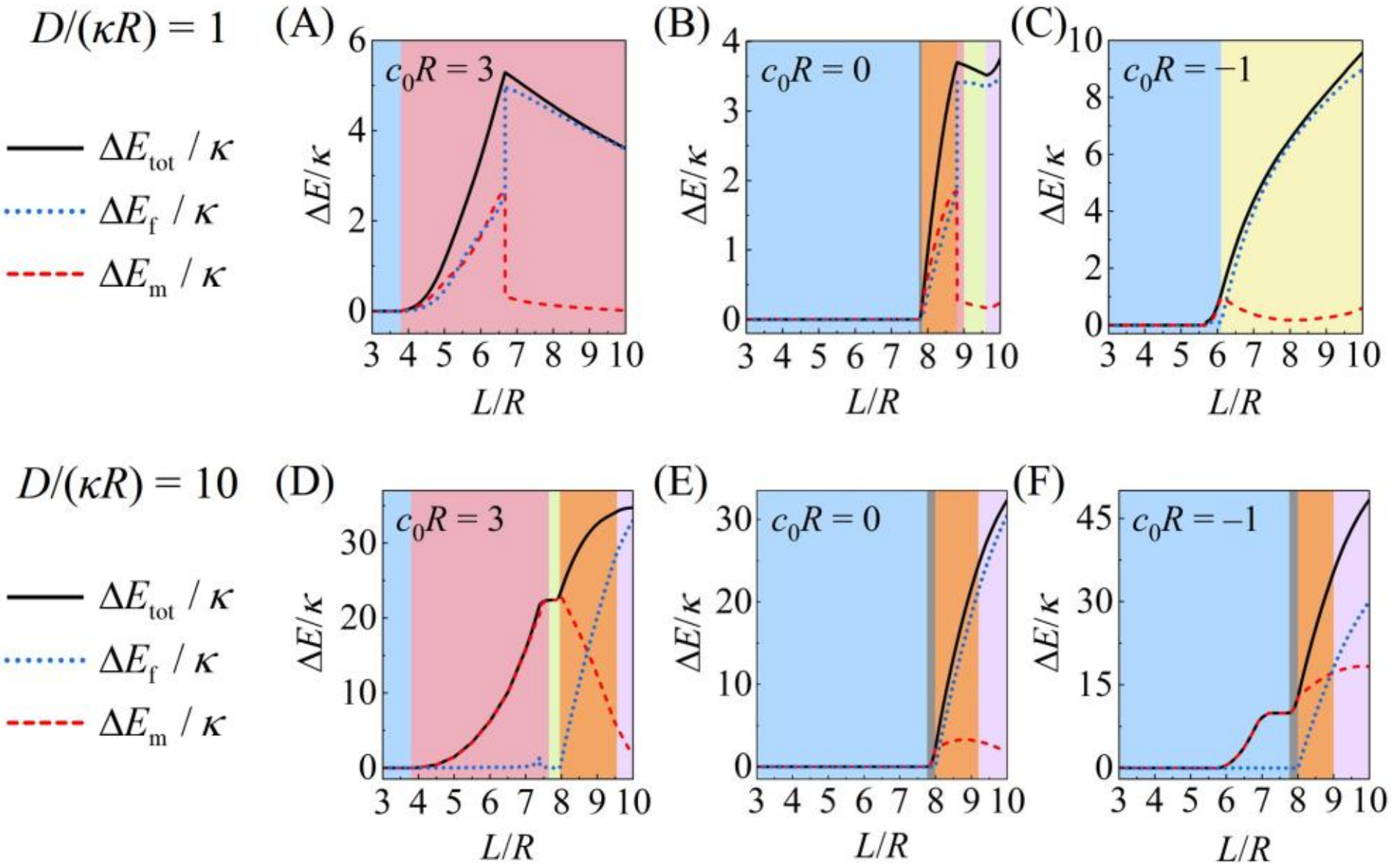

Figure S7. Profiles of the total elastic energy change ΔE_{tot} and its components ΔE_{f} and ΔE_{m} at v = 0.65. Panels (A–C) correspond to $D/(\kappa R)$ = 1 and panels (D–F) to $D/(\kappa R)$ = 10.

Complementing the phase diagrams at v = 0.65, Figure S7 presents the total energy change ΔE_{tot} along with its membrane and filament contributions, ΔE_{m} and ΔE_{f}, for flexible ($D/(\kappa R)$ = 1) and stiff ($D/(\kappa R)$ = 10) filaments. The reference membrane energy E_{m0} is 1.41κ, 45.90κ, and 68.38κ at c_0R = 3, 0, and −1, respectively. For flexible filaments (Figure S7A–C), at intermediate filament lengths, ΔE_{m} and ΔE_{f} contribute comparably for c_0R = 3 and 0, whereas ΔE_{m} dominates at c_0R = −1; at large L, ΔE_{f} dominates in all cases. For stiff filaments (Figure S7D–F), ΔE_{m} dominates at short and intermediate L for c_0R = 3, whereas ΔE_{f} prevails at large L; at c_0R = 0, ΔE_{f} dominates throughout, and at c_0R = −1, ΔE_{m} governs the intermediate-L regime while both ΔE_{m} and ΔE_{f} remain important at large L.

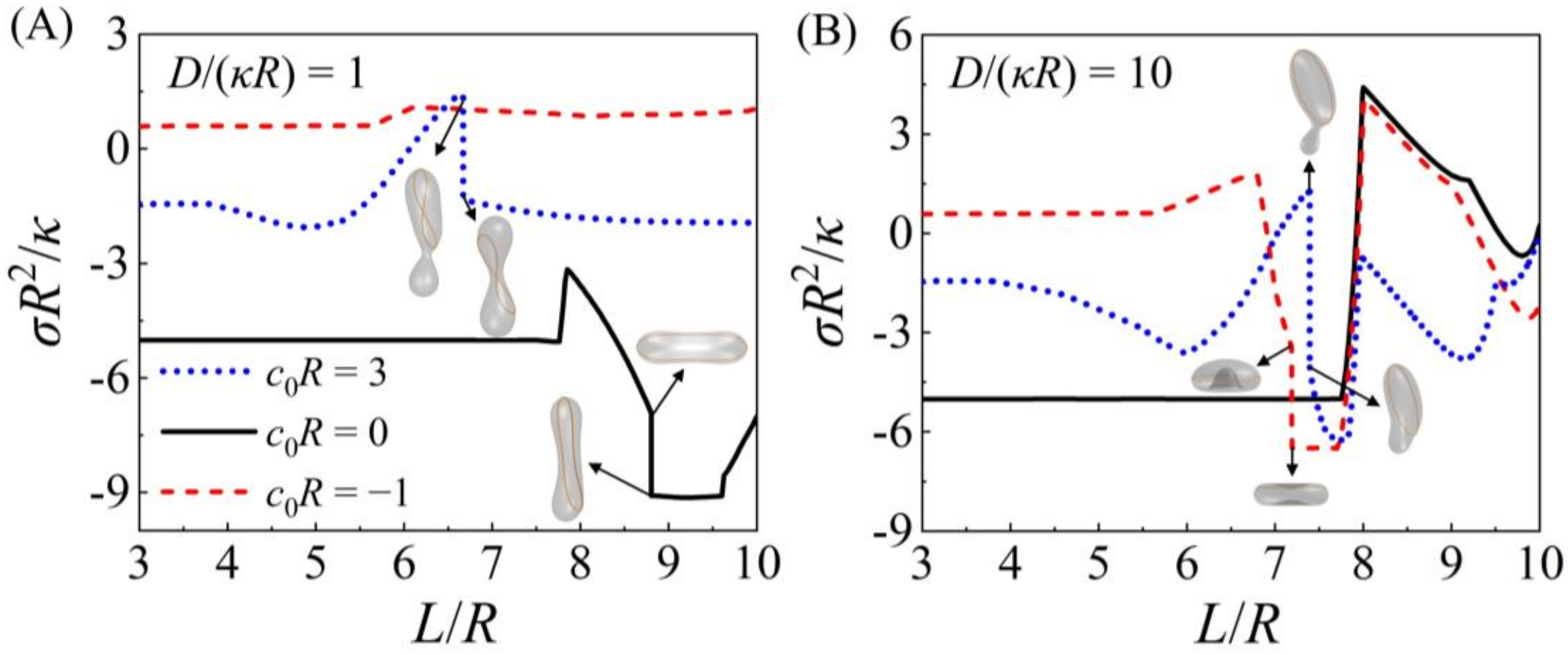

Figure S8. Profiles of the normalized membrane tension $\sigma R^2/\kappa$ at v = 0.65 for filaments with $D/(\kappa R)$ = 1 (A) and 10 (B). In each panel, curves are shown for c_0R = −1, 0, and 3. Insets show representative transition configurations.

Figure S8 provides the membrane tension evolution as a function of filament length. For flexible filaments ($D/(\kappa R)$ = 1), an abrupt drop in σ at c_0R = 3 (dotted blue line) indicates a discontinuous transition in which the filament escapes single-lobe confinement to span the vesicle waist. At c_0R = 0, σ first exhibits a sharp increase associated with a continuous $C_{\infty v}$-to-$D_{\infty h}$ transition, followed by a sudden decrease due to the discontinuous D_{2h}-to-C_s transition. At c_0R = −1, the filament primarily accommodates stomatocyte confinement through bending, resulting in only weak tension variations.

For stiff filaments ($D/(\kappa R)$ = 10), the tension variations are more pronounced. At c_0R = 0, σ rises sharply during the continuous $C_{\infty v}$-to-$D_{\infty h}$ transition. At c_0R = 3 and −1, abrupt drops in σ indicate discontinuous transitions that relieve membrane tension, corresponding to waist opening and the stomatocyte-to-oblate transformation, respectively. The subsequent tension evolution converges to a common continuous trajectory characterized by the D_{2h}-to-D_2 transition, regardless of c_0R.

Note 3. Shared configuration data

The MATLAB .fig files provided as Supporting Data contain representative optimized filament–vesicle configurations for visualization and inspection. Each file can be opened in MATLAB and rotated freely to examine the corresponding vesicle surface and filament-loop geometry. The plotted objects can also be used to extract representative geometric information from the displayed configurations.

The files are named using the format v[value]_NormL[value]_c0R[value]_SR[value].fig, where v is the reduced volume, NormL denotes the normalized filament length L/R, c0R denotes the normalized spontaneous curvature c_0R, and SR denotes the stiffness ratio $D/(\kappa R)$. The corresponding parameter values are summarized in Table S2.

Table S2. MATLAB .fig files and corresponding model parameters.

**Shared .fig file**	***v***	***L/R***	**c_0R**	**$D/(\kappa R)$**
v0.9_NormL4.5_c0R1_SR1.fig	0.9	4.5	1	1
v0.9_NormL5.6_c0R2.5_SR10.fig	0.9	5.6	2.5	10
v0.9_NormL7.475_c0R-2_SR10.fig	0.9	7.475	−2	10
v0.9_NormL9_c0R2.4_SR1.fig	0.9	9	2.4	1
v0.65_NormL3.4_c0R2_SR1.fig	0.65	3.4	2	1
v0.65_NormL5.6_c0R-0.8_SR1.fig	0.65	5.6	−0.8	1
v0.65_NormL7.1_c0R-1_SR10.fig	0.65	7.1	−1	10
v0.65_NormL7_c0R1.5_SR10.fig	0.65	7	1.5	10
v0.65_NormL8_c0R-0.5_SR1.fig	0.65	8	−0.5	1
v0.65_NormL9.2_c0R3_SR1.fig	0.65	9.2	3	1
